# Chylous Leakage After Breast-Conserving Surgery and Axillary Clearance: Case Report and Management Strategies

**DOI:** 10.3389/fonc.2022.878645

**Published:** 2022-03-29

**Authors:** Liang Yin, Peiqing Chen, Enock Adjei Agyekum, Xiudi Xiao, Xiaoqin Qian

**Affiliations:** ^1^ Department of Breast Surgery, Jiangsu University Affiliated People’s Hospital, Zhenjiang, China; ^2^ Department of Ultrasound, Jiangsu University Affiliated People’s Hospital, Jiangsu University, Zhenjiang, China

**Keywords:** chylous leakage, breast cancer, breast-conserving, axillary clearance, case report management strategies

## Abstract

Chylous leakage is a rare complication of breast and axillary surgery. We present a case of chylous leakage inside the breast following breast-conserving surgery and axillary lymph node dissection. The majority of chylous leakages in the breast are managed with conservative measures aimed at reducing lymphatic fluid production and outflow. Surgical intervention is required in cases of conservative treatment failure and high output chylous leakage. To the best of our knowledge, this is the first case report of chyles leaks inside the breast following breast-conserving surgery that was successfully treated surgically.

## Introduction

Chylous leakage is a well-known complication of the neck, thoracic, and upper gastrointestinal surgery. Its incidence ranges from 0.5% to 8.3% in neck dissection, with the majority of cases occurring on the left side due to thoracic duct injury (1, 2). However, its occurrence following breast and axillary surgery is a rare occurrence. In breast cancer surgery, the reported incidence ranges from 0.36% to 0.84% in the literature (3). Given the exceedingly rare occurrence, there is currently little guidance on the diagnosis and management of chylous leakage. We present a case of chylous leakage after breast-conserving surgery and axillary clearance in a patient with solid papillary carcinoma of the right breast.

## Case Report

A 67-year-old woman came to our hospital complaining of a lump in her right breast. An ultrasound of the breast revealed a 2.5*2-cm solid mass in the upper outer quadrant of the right breast, with no enlarged right axillary lymph node. Mammography revealed a high density, ill-defined lump in the upper outer quadrant of the right breast. The patient was diagnosed with stage IIA breast cancer. We decided to proceed with breast-conserving surgery, namely a lumpectomy and sentinel lymph node biopsy using methylene blue injection through a single incision, after discussing treatment options with the patient. During the operation, the rapid freezing pathology revealed solid papillary carcinoma with a negative surgical margin. On frozen, one of three sentinel lymph nodes tested positive for malignancy, necessitating level II axillary lymph node dissection. In the axillary and breast cavity, a single drain was placed. Histopathological examination revealed a highly differentiated lumina A and 25mm solid papillary carcinoma in the right breast. A total of 16 lymph nodes were removed. One of them was found to have tumor metastasis. The patient was given endocrine therapy in the form of 1 mg of anastrozole orally every day.

**Figure 1:**
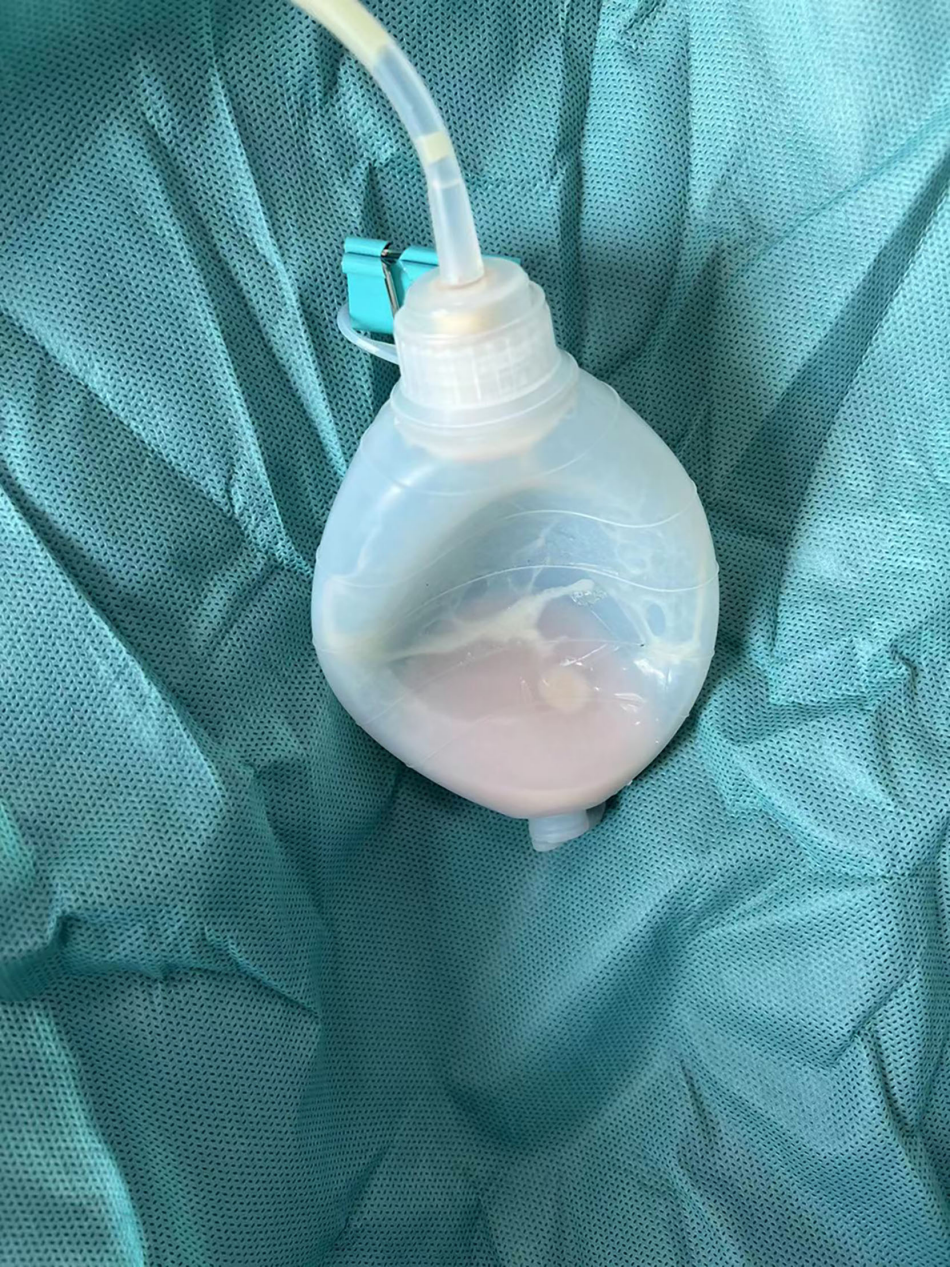
After percutaneous drainage, chylous fluid was present in bulb.

Her postoperative recovery went smoothly. The drain was removed on postoperative day (POD) 5, and the patient was discharged on POD 7. On POD 10, she presented to our facility with a slight swelling of the axilla. By puncturing the axillary cavity, 20 milliliters of a milky fluid were aspirated. Initially, we tried conservative treatment such as closed suction drainage, a compressive bandage, and a low-fat diet. A biochemistry analysis of the drainage fluid revealed 1201 mg/dL of triglycerides, and the celiac test was positive. However, the daily output of the drain was kept at around 200ml for two months (**Figure 1**). Following the failure of conservative management, the patient refused radiotherapy. After consulting with the patient, we decided to perform exploration and mastectomy. The clear fluid was observed to be coming from a single duct located in the breast surgery bed rather than the axillary cavity. The duct was ligated, and the breast was removed. The incision was closed after one drain was placed. Drain output dropped to 80ml/d after the re-operation, and it remained slightly milky for the next 10 days. The drain was removed, and there has been no evidence on a regular diet.

## Discussion

Because of the anatomically more remote position of the thoracic duct, chylous leakages are uncommon after breast and axillary surgery. However, as the results show, chylous leaks are not limited to the left side. Thoracic duct anatomical variants are well documented in the literature (4). This is not surprising given that only 50% of people have the typical anatomy. The duct may empty on the right in 2-3% of cases, and bilateral emptying occurs in 1.5% (5). Furthermore, the type of axillary procedure used may play a role in determining which patients will experience chylous leakage. Because the duct collapses after injury, it is difficult to recognize lymphatic duct injuries intraoperatively. Because of the rarity of lymphatic trunk injury and the lack of well-known risk variables, it is also difficult to predict injury to the lymphatic trunks preoperatively.

To the best of our knowledge, this is the first and only case of celiac leakage in the breast following breast-conserving surgery and axillary lymph node dissection. David T Pointer Jr described a case of chyle leak after breast-conserving surgery and sentinel lymph node biopsy. The celiac leakage was discovered at the site of a sentinel lymph node biopsy rather than in the breast (6). The other cases occurred after mastectomy and axillary lymph node dissection, which had more extensive surgery than our presented case (7, 8).

Chylous leakage is typically diagnosed when a milky white fluid drains from the surgical drain. Biochemical testing of the fluid’s electrolyte, protein, and lipid content, all of which are compatible with chyle in these cases, confirms the definitive diagnosis. Lymphoscintigraphy or computed tomography is a useful tool for locating chyle fistulas and confirming chyle collection.

The majority of chylous leakages respond to conservative management. To avoid the formation of a collection, a low-volume leak can be handled simply by draining and monitoring. Negatively pressured drainage and free drainage were described in the literature, and the use of pressure bandaging in conjunction with drainage was also mentioned (9). Local injection of hypertonic glucose or meglumine diatrizoate was thought to be an effective treatment for refractory chylous leakage, because drugs can cause aseptic inflammation, resulting in lymphatic vessel closure (10, 11). Dietary fats are known to increase chyle volume, a low-fat diet may help to reduce flow volumes and allow damaged lymphatic capillaries to repair. As a result, in primary conservative management, a diet rich in medium-chain triglycerides (MCT) or parenteral nutrition support is recommended (12). Some authors advocate the use of octreotide to reduce chylous output by inhibiting gastrointestinal motility and secretions (13).

Surgical intervention of chyle leak, on the other hand, has been discussed in a number of studies (6, 14, 15). Some authors believe that early surgical intervention may be beneficial in patients who have failed to respond to initial dietary and/or medical interventions. Because the risk of re-exploration of the axilla and breast is low, and earlier chylous fistula ligation can prevent subsequent oncologic treatments from being delayed, the damaged lymphatic channel is directly ligated during surgery. Intraoperative orogastric or nasogastric boluses of “heavy cream,” as demonstrated by Pointer and colleagues, can aid in the identification of the leaking vessel (6). As an alternative, plugging with gel foam, adhesive, local muscle rotation flaps, or other packing materials could be considered.

## Conclusion

Chylous leakage following breast conserving surgery and axillary clearance is a rare but significant complication. The majority of chylous leakage occurs during axillary surgery; however, we should be aware of the possibility of chylous leakage during breast surgery as well. Individualized management of chylous leakage following breast and axillary dissection is required. Early surgical intervention is recommended for conservative treatment failure and high output fistulas.

## Data Availability Statement

The original contributions presented in the study are included in the article/supplementary material. Further inquiries can be directed to the corresponding author.

## Ethics Statement

The studies involving human participants were reviewed and approved by the Ethics Committee of Jiangsu University Affiliated People’s Hospital. The patients/participants provided their written informed consent to participate in this study. Written informed consent was obtained from the individual(s) for the publication of any potentially identifiable images or data included in this article.

## Author Contributions

All authors listed have made a substantial, direct, and intellectual contribution to the work and approved it for publication.

## Funding

Research project of Jiangsu Maternal and Child Health Association (FYX202004). This study was financially supported by National Natural Science Foundation of China (Project No.:81971629) and Research Project of Jiangsu Maternal and Child Health Association (Grant No. FYX202004).

## Conflict of Interest

The authors declare that the research was conducted in the absence of any commercial or financial relationships that could be construed as a potential conflict of interest.

## Publisher’s Note

All claims expressed in this article are solely those of the authors and do not necessarily represent those of their affiliated organizations, or those of the publisher, the editors and the reviewers. Any product that may be evaluated in this article, or claim that may be made by its manufacturer, is not guaranteed or endorsed by the publisher.
